# TREATMENT OF PATIENTS WITH GIANT CELL BONE TUMOR IN NORTHERN BRAZIL, IN 2020 AND 2021

**DOI:** 10.1590/1413-785220253301e285342

**Published:** 2025-02-03

**Authors:** Fernando Brasil do Couto, Eduardo Sadao Yonamine, Felipe Guimarães Magno, Ana Beatriz Favacho-Silva, Carlos Rafael Alves de Brito, Thiago Raphael Brasil Brito

**Affiliations:** 1 Hospital Ophir Loyola, Orthopaedic Oncology Department, Belém, PA, Brazil; 2 Santa Casa de Misericórdia de São Paulo, Orthopaedic Oncology Department, São Paulo, SP, Brazil

**Keywords:** Giant Cell Tumors, Cohort Studies, Bone Neoplasms, Tumores de Células Gigantes, Estudos de Coortes, Neoplasias Ósseas

## Abstract

**Objective:**

This study aimed to evaluate the profile of patients diagnosed with giant cell tumors treated at the Ophir Loyola Hospital.

**Method:**

An analytical study in the form of a retrospective cohort, conducted through a review of patient medical records and imaging exams of individuals treated at the hospital between January 1, 2020, and December 31, 2021.

**Result:**

A total of 19 patients were evaluated, with an average time of 10.5 months between diagnostic suspicion and the first consultation with the Orthopedic Oncology team.

**Conclusion:**

Among the patients studied, 73% were presented with advanced-stage disease, requiring aggressive surgical treatment with wide resection and replacement with an endoprosthesis. ***Level of Evidence II; Retrospective Prognostic Study.***

## Resumo

**Objetivo:**

Avaliar o perfil do paciente com diagnóstico de tumor de células gigantes atendidos no Hospital Ophir Loyola.

**Método:**

Estudo analítico em forma de coorte retrospectiva, realizado por revisão dos prontuários e exames de imagem dos pacientes atendidos no hospital no período de 1 de janeiro de 2020 a 31 de dezembro de 2021.

**Resultado:**

Foram avaliados 19 pacientes, com tempo médio de 10,5 meses entre a suspeita diagnóstica e a primeira consulta com a equipe da Oncologia Ortopédica.

**Conclusão:**

Dos pacientes estudados, 73% apresentavam-se em estágio avançado, necessitando tratamento cirúrgico agressivo com ressecção ampla e substituição por endoprótese. **Nível de Evidência II; Estudo Prognóstico de Caráter Retrospectivo.**

## INTRODUCTION

Giant cell tumors (GCT) are rare, accounting for less than 10% of benign primary bone tumors. They have a higher prevalence after skeletal maturity, although they can also be found in patients with open growth plates. It is considered one of the most controversial and least predictable tumors in its behavior.^1^,^2^

They preferably affect long bones, although they have been reported in the spine, pelvis, patella, and sacrum. They are differentially diagnosed from aneurysmal bone cyst, chondroblastoma, and simple bone cyst.^3^

The main clinical symptoms are pain and swelling. Diagnoses can be suspected through a pathological fracture as the initial presentation. Imaging tests such as X-rays, often show the triad: epiphyseal, eccentric, and lytic lesions. The bone cortex may show thinning or local destruction. The transition zone is not well defined, but in less aggressive cases, there is sclerotic reaction. Invasion and destruction of the cortex with invasion of soft tissues are commonly observed. Sometimes there is no joint involvement due to the barrier formed by the subchondral bone.^4^,^5^

The interval between the first symptom and the definitive diagnosis is termed as “delay time”. The period between the initial symptoms and the initial treatment can be divided into two main categories: patient delay, which is defined as the time between the initial symptoms and the first medical consultation, and professional delay, primarily caused by the physician, defined as the time between the first consultation and the initial treatment. The addition of these two delays is called the overall symptom interval.^6^

Diagnosis is made through biopsy, and treatment is surgical, which may involve intralesional resection with curettage, adjuvants and/or cavity filling with bone cement, auto or allograft; in more advanced cases, wide resection with endoprosthesis replacement or even amputation may be performed.^2^,^7^

Since the morbidity of these patients is directly related to the time between diagnosis and treatment, it is important to understand the patient entry profile into the service and the outcomes of their treatment. Furthermore, there are no similar studies in the Amazon region.

To address the issues, this study aims to evaluate the profile of patients diagnosed with GCT treated at Hospital Ophir Loyola in the Years 2020 and 2021, demonstrating the time between diagnostic suspicion and the first consultation with the Orthopedic Oncology team, the characteristics of the lesion at the initial consultation and the proposed treatment.

## METHODOLOGY

This is an analytical study, in the form of a retrospective cohort, which will be conducted through the analysis of medical records and imaging studies of patients seen in the outpatient clinic from January 1, 2020, to December 31, 2021, at a quaternary hospital. The study includes 19 patients diagnosed with GCT treated at the Orthopedic Oncology outpatient clinic from January 1, 2020, to December 31, 2021. The medical records of patients seen in the years 2022 and 2023 were not analyzed due to data unavailability at the service until the study was completed.

Patients with suspected diagnosis but who did not undergo biopsy were excluded from the study.

The research was conducted through medical record review and analysis of imaging studies.

The studied variables were divided into demographic and clinical. Demographic variables include age, sex, and place of origin. Clinical variables include the date of diagnostic suspicion, the date of the first consultation with the Orthopedic Oncology team, the date of diagnosis, the anatomical location of the tumor, the tumor stage, the presence of pulmonary metastasis, the presence of recurrence, date of pathological fracture (if applicable), type of surgery, type of cavity filling (if applicable), adjuvants used (if applicable), and the use and indication of denosumab. First, the medical records of patients seen in the outpatient clinic with ICD-10 D16 were selected to screen for patients diagnosed with GCT. Then, date contained in the medical records and Imaging studies database were collected. A questionnaire was filled out for standardization purposes.

A descriptive analysis of the sample characterization was conducted, with frequencies, percentages, mean, standard deviation, median, interquartile range (p25%-p75%), presented in tables and/or graphs. Continuous quantitative variables, such as age (years) and duration of suspicion (months), were first subjected to the Shapiro-Wilk test to analyze their normal distribution.

For comparative analysis between the stage and recurrence groups, the Mann-Whitney test was Applied for continuous variables. The Fisher’s Exact Test was used for comparative analysis between categorical variables.

All statistical analyses were performed using SPSS 20.0 software respecting a significance level of 5% (p≤0.05).

This research was approved by the Institutional Ethics Committee through the Plataforma Brazil, with identification number CAEE 7509023.6.0000.5550 (amendment no. 6.585.557). The administration of questionnaires and retrospective medical record collection were preceded by a Data Use Agreement to ensure the reliability of the collected information, and a request for Waiver of Informed Consent was submitted.

## RESULTS

The initial database included 137 patients. After reviewing the medical records, patients diagnosed with GCT through biopsy were selected. Thus, the final database consisted of 19 patients treated at the Orthopedic Oncology outpatient clinic in the years 2020 and 2021. The majority of patients were male (57.89%); with mean age of 37.32 (±13.47) years; residents of other regions of the state of Pará (47.37%), outside the Metropolitan region of Belém; with a mean patient delay time of 10.47 (±13.08) months; predominantly with lesions located in the distal femur region (47.37%), at Campanacci stage 3 (73.68%); without a history of pathological fracture (84.21%); undergoing mostly marginal/wide surgical type (73.68%) and without a history of recurrence (84.21%), as detailed in Table 1.

**Table 1:** Analysis of the demographic and clinical profile of patients diagnosed with giant cell tumor treated at a quaternary hospital in Northern Brazil, in the years 2020 and 2021.

Variable	Frequency (n. 19)	Percentage (%)	CI95%
**Sex**			
Male	11	57.89	36.8 - 78.9
Female	8	42.11	21.1 - 63.2
**Age (years)**			
Mean ( sd)	37.32 ( 13.47)		31.0 - 43.8	
Median (p25-75%)	39.00 (24.00 - 45.50)		24.0 - 44.0	
**Age range**			
< 30 years	6	31.58	10.5 - 52.6
≥ 30 years	13	68.42	47.4 - 89.5
**Location**			
Metropolitan region of Belém	6	31.58	10.5 - 52.6
Other regions of Pará	9	47.37	26.3 - 68.4
Other state	4	21.05	5.3 - 42.1
**Patient delay (months)**			
Mean ( sd)	10.47 ( 13.08)		6.1 - 17.1	
Median (p25-75%)	7.00 (4.00 - 12.00)		4.0 - 12.0	
**Patient delay ≥ 12 months**			
Yes	6	31.58	10.5 - 52.6
No	13	68.42	47.4 - 89.5
**Anatomic location**			
Proximal femur	1	5.26	0.0 - 15.8
Distal femur	9	47.37	21.2 - 68.4
Proximal tibia	6	31.58	10.5 - 52.6
Calcaneus	1	5.26	0.0 - 15.8
Hand bones	2	10.53	0.0 - 26.3
**Campanacci**			
Stage 1	2	10.53	0.0 - 26.3
Stage 2	3	15.79	0.0 - 31.6
Stage 3	14	73.68	52.5 - 89.5
**Pathological fracture**			
Yes	3	15.79	0.0 - 36.8
No	16	84.21	63.2 - 100.0
**Surgery type**			
Wide resection	14	73.68	52.5 - 89.5
Marginal/Intralesional	3	15.79	0.0 - 31.6
Not performed	2	10.53	0.0 - 26.3
**Recurrence**			
Yes	3	15.79	0.0 - 36.8
No	16	84.21	63.2 - 100.0
**Denosumab use**			
Yes	5	26.32	10.5 - 47.4
No	14	73.68	52.5 - 89.5
**Pulmonary metastasis**			
Yes	0	0.00	0.0 - 0.0
No	19	100.00	100.0 - 100.0

Sd: Standard deviation. P: Percentile. CI: confidence interval.

The most employed treatment among patients in this study was surgery with wide resection and replacement with unconventional endoprosthesis. Only 2 patients did not undergo treatment at the hospital due to loss to follow-up; for one male patient with GCT in the calcaneus, intralesional resection with cavity filing was requested, and for one female patient with GCT in the distal third of the femur, wide resection and replacement with unconventional endoprosthesis were requested, both patients did not attend for hospitalization.

Three patients experienced tumor recurrence after primary treatment. Two patients experienced recurrence about 2 years after surgery with wide resection and replacement with unconventional endoprosthesis, and they were managed with denosumab prescription. The third patient was diagnosed with tumor recurrence 6 months after surgical treatment, also with wide resection and unconventional endoprosthesis, denosumab was prescribed without significant improvement, progressing to transfemoral limb amputation.

Although not planned criteria, during the research, complications were noted in 4 patients undergoing surgical treatment. One patient initially treated with intralesional resection and osteosynthesis required endoprosthesis replacement due to significant worsening of knee osteoarthritis after initial surgical treatment. Two patients required surgery revision due to dislocation or loosening of components. And one patient progressed to transfemoral limb amputation due to surgical site infection.

None of the patients in this study were diagnosed with pulmonary metastasis.

When comparing demographic and clinical differences between the Campanacci stages groups, it was observed that patients in Stage 3 differed significantly (p-value: 0.002) from Stages 1 and 2 regarding the type of surgery performed. While in stages 1 and 2, the most used surgery type was intralesional or marginal (60%), in the stage 3, wide surgery stood out (92.9%). The other variables did not differ significantly, as detailed in Table 2.

**Table 2:** Comparative analysis of the demographic and clinical profile of patients diagnosed with GCT in Stages 1/2 and Stage 3 treated at a quaternary hospital in Northern Brazil, in the years 2020 and 2021.

Variable	Stage 1 e 2 (n. 5)	Stage 3 (n. 14)	p-value
**Sex**			
Male	1(20.0%)	10(71.4%)	0.071^a^
Female	4(80.0%)	4(28.6%)	
**Age(years)**			
Mean( sd)	38.6( 9.6)	36.8( 14.9)	0.830^b^
Median(p25-75%)	39.0(39.0-43.0)	36.5(24.0-47.0)	
**Agerange**			
<30years	1(20.0%)	5(35.7%)	0.480^a^
>30years	4(80.0%)	9(64.9%)	
**Location**			
MetropolitanregionofBelém	1(20.0%)	5(35.7%)	0.814^a^
OtherregionsofPará	3(60.0%)	6(42.9%)	
Otherstate	1(20.0%)	3(21.4%)	
**Patientdelay**			
Mean( sd)	14.0( 25.7)	9.2( 5.2)	0.070^b^
Median(p25-75%)	3.0(2.0-4.0)	7.5(6.0-12.0)	
**Patientdelay≥12months**			
Yes	1(20.0%)	5(35.7%)	0.631^a^
No	4(80.0%)	9(64.3%)	
**Anatomiclocation**			
Proximalfemur	0(0.0%)	1(7.1%)	0.164^a^
Distalfemur	3(60.0%)	6(42.9%)	
Proximaltibia	0(0.0%)	6(42.9%)	
Calcaneus	1(20.0%)	0(0.0%)	
Handbones	1(20.0%)	1(7.1%)	
**Pathologicalfracture**			
Yes	0(0.0%)	3(21.4%)	0.530^a^
No	5(100.0%)	11(78.6%)	
**Surgerytype**			
Wideresection	1(20.0%)	13(92.9%)	0.002^a^*
Marginal/Intralesional	3(60.0%)	0(0.0%)	
Notperformed	1(20.0%)	1(7.1%)	
**Recurrence**			
Yes	0(0.0%)	3(21.4%)	0.530^a^
No	5(100.0%)	11(78.6%)	
**Denosumabuse**			
Yes	0(0.0%)	5(35.7%)	0.257^a^
No	5(100.0%)	9(64.3%)	
**Pulmonarymetastasis**			
Yes	0(0.0%)	0(0.0%)	1.000^a^
No	5(100.0%)	14(100.0%)	

Sd: Standard deviation. P: percentile a: Fisher’s exact test. B: Mann-Whitney test. *. p-value < 0.05.

## DISCUSSION

To better understand the profile of patients diagnosed with GCT seen at our service, we noted that the average time between diagnostic suspicion and consultation with the Orthopedic Oncology team was 10.5 months, with the tumor being in an advanced stage, necessitating extensive surgical treatment.

Among all patients seen in the service during the study period, those diagnosed with GCT presented a prevalence of approximately 14%. This is above the average described in the literature, but it is important to consider that the initial sample does not solely account for bone tumors.^8^,^9^

Despite epidemiological studies showing a slight predominance among females, most of our patients were male.^8^,^9^,^10^

The average range of most patients, the primary site of the tumor, and the presence of pathological fractures in our study are consistent with epidemiological studies. At diagnosis, most patients were between 20 and 39 years old, a finding similar to the literature.^11^,^12^ The most common primary sites were the distal third of the femur and the proximal third of the tibia, at 47% and 31%, respectively. The knee is the most common primary site in the body, with the distal third of the femur being the main site, followed by the proximal third of the tibia. When it affects, the distal third of the radius, the third most common site described in the literature, it usually exhibits more evident aggressive characteristics. Involvement of the sacrum is rare.^8^,^9^

Our service recorded the bones of the hand as the third most common site. The fact that 10% of tumors were in the hand differs from the epidemiology found in the literature. In 2021, a study demonstrated the low prevalence of giant cell tumors in the phalanges. In a study involving 2.400 patients, there were fewer than 50 cases, while another study showed only 1 case among 327 patients.^13^

Pathological fractures occurred in 3 patients, corresponding to 15% of the group. Most patients experience progressive pain, initially manifesting during activities but progressing to rest pain. It is not usually disabling, except in cases associated with pathological fractures which are evident in the initial examination of 10 to 30% of patients.^14^

**Figure 1:**
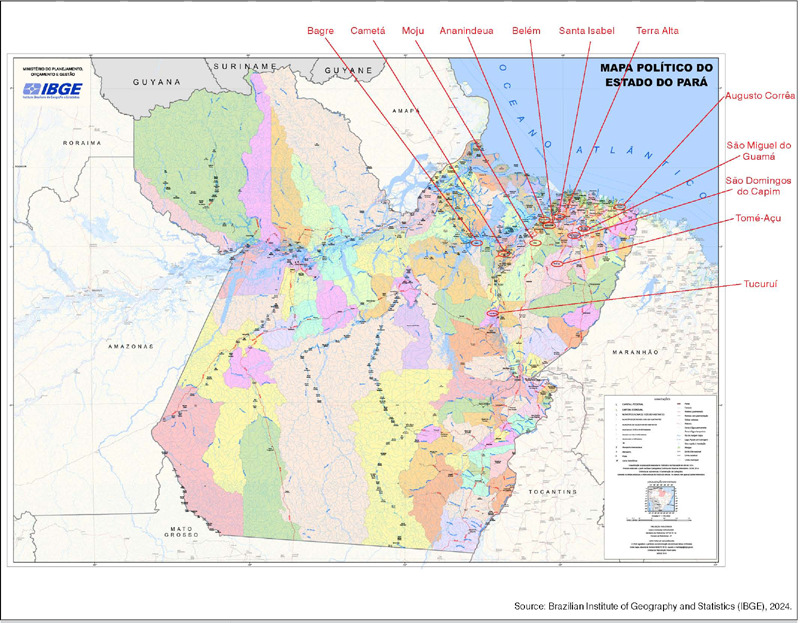
Political map of the state of Pará.

Source: Brazilian Institute of Geography and Statistics (IBGE), 2024.

The average diagnosis delay time is considered high, which directly influences the increased likehood of diagnosis tumors at advanced stages.^15^ The average patient delay time of 10.5 months may reflect the size of the state of Pará, the difficulties faced by the Public Health Service in identifying the need for specialized care early on and may also indicate difficulties in accessing health services for the more deprived population in remote areas. Additionally, there were patients from the state of Maranhão receiving treatment, showing that the complexity of health problems extends nationally (Figure 1 2).

**Figure 2:**
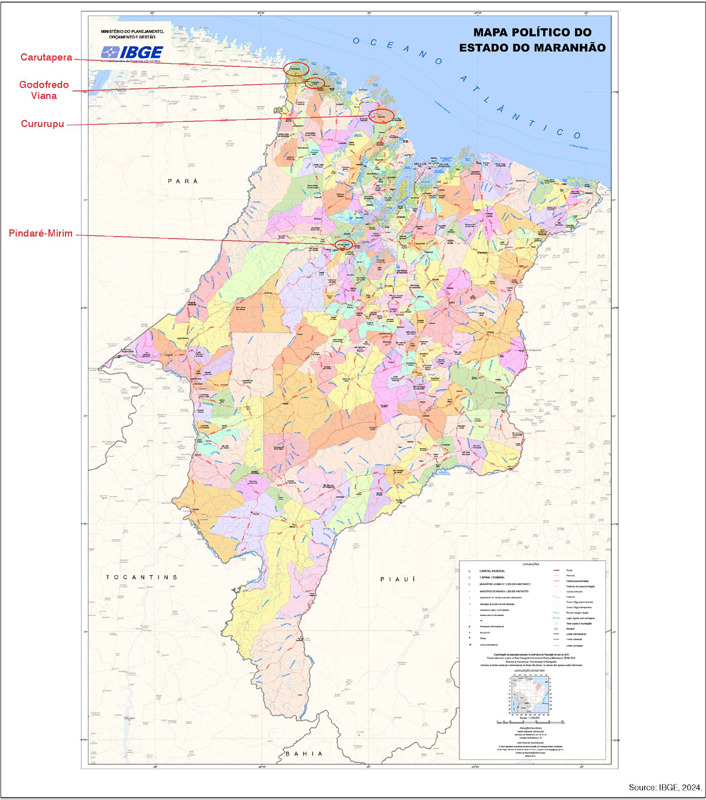
Political map of the state of Maranhão.

Source: IBGE, 2024.

Currently, in our service the increase in the Orthopedic Oncologist team in 2023, there is no longer a surgical procedure waiting list, as was the reality in the period of 2020 and 2021. There is now a window of only a few weeks between the indication for surgical treatment and the procedure. However, the main difficulty lies in the stage at which patients arrive for the initial consultation. Over 70% of patients presented at Campanacci stage 3, highlighting the diagnostic delay we face in the state and the direct relationship between Campanacci Stage 3 and the average time of 10.5 months for diagnosis.^15^,^16^

Due to this, the primary surgical treatment employed in these patients was wide resection with replacement with unconventional endoprosthesis, differing from much of the national and worldwide literature, which shows that most diagnosed patients are in Campanacci stage 2 and are managed with intralesional resection and cavity grafting.^17^ Even with aggressive treatment, there was still a case requiring amputation due to recurrence, highlighting the importance of early diagnosis as a major modulator in the prognosis of these patients.

Studies over the last decade have already demonstrated the relationship between early diagnosis and the severity of the lesion, as well as the temporal relationship between symptom onset and tumor severity. Early diagnoses reduce morbidity related to both diagnosis and surgical treatment, in addition to bringing benefits to the public health system.^15^

Recurrences are defined on symptomatic evidence or changes in imaging studies. They are considered from three months after treatment, but can be detected within the first two years.^8^ The rate of local recurrence can be influenced by diagnostic delay and the surgical technique employed. Campanacci 1 and 2 are treated with curettage and adjuvants. Isolated intralesional surgeries have a recurrence risk of 50%, decreasing to 30% with the use of local adjuvants. Campanacci 3 is generally treated with radical excision due to the high risk of recurrence, often requiring joint reconstruction with endoprosthesis. The risk of recurrence after this therapeutic modality is around 0-12%. The recurrence rate in our study was 15% with the need for denosumab treatment or limb amputation.^8^,^10^,^18^

In our service, denosumab treatment was used before surgical approach in 2 patients. In another 3, was used after the diagnosis the tumor recurrence. The medication is a biological agent that prevents bone cell destruction by interrupting osteoclast maturation, as it prevents RANK activation by binding to RANK-L. It is indicated for use in cases where it is not possible to completely excise the tumor, which would increase the risk of recurrence, and in cases where surgery is contraindicated. 18,19 The use of medication does not cure the disease, but has a limb-preserving potential, preventing amputations, hemipelvectomy, neurological disorders, and reducing morbidity associated with surgical treatment.^19^,^20^

As mentioned earlier, several factors influence the diagnostic delay of oncology patients. The hospital serves as a reference for the entire state, and the demographic and socioeconomic constraints in the state of Pará are significant and impact the reality of public health. We must also consider the knowledge of generalist physicians and clinical areas regarding bone tumors because often these professionals will provide initial care. Recognizing an aggressive tumor and knowing that referral to a specialized service is necessary directly impact the early treatment of these patients.

Our study showed an average time of 10.5 months between diagnostic suspicion and the first consultation with the Orthopedic Oncologist. At the time of diagnosis, 73% of patients presented with Giant Cell Tumor in an advanced stage, Campanacci grade 3, necessitating aggressive surgical treatment with wide resection and endoprosthesis replacement.
